# Human Bocavirus and KI/WU Polyomaviruses in Pediatric Intensive Care Patients

**DOI:** 10.3201/eid1503.081203

**Published:** 2009-03

**Authors:** Alma C. van de Pol, Tom F.W. Wolfs, Nicolaas J.G. Jansen, Jan L.L. Kimpen, Anton M. van Loon, John W.A. Rossen

**Affiliations:** University Medical Center Utrecht, Utrecht, the Netherlands (A.C. van de Pol, T.F.W. Wolfs, N.J.G. Jansen, J.L.L. Kimpen, A.M. van Loon, J.W.A Rossen); St. Elisabeth Hospital Tilburg, Tilburg, the Netherlands (J.W.A. Rossen)

**Keywords:** Human bocavirus, polyomaviruses, children, respiratory tract infections, dispatch

## Abstract

We evaluated the prevalence of human bocavirus and KI and WU polyomaviruses in pediatric intensive care patients with and without lower respiratory tract infection (LRTI). The prevalence of these viruses was 5.1%, 0%, and 2.6%, respectively, in children with LRTI and 4.8%, 4.8%, and 2.4%, respectively, in those without LRTI.

Through use of molecular diagnostic tests such as real-time PCR in the clinical setting, our scope of etiologic viral agents of lower respiratory tract infection (LRTI) has increased. Respiratory viruses can now be detected in most pediatric intensive care patients with LRTI (*1*). Recently, 3 new viruses were described: human bocavirus (HBoV) and KI (KIPyV) and WU (WUPyV) polyomaviruses (*2*–*4*). These viruses were first identified in respiratory samples obtained from children with respiratory tract infections. An association between the viruses and respiratory tract symptoms was postulated, but, to date, evidence supporting that association is incomplete (*5*–*7*). This study evaluates the prevalences of HBoV, KIPyV, and WUPyV infections in pediatric intensive care patients with acute respiratory insufficiency caused by LRTI.

## The Study

Patients <5 years of age who were admitted for LRTI to the pediatric intensive care unit (PICU) of Wilhelmina Children’s Hospital, Utrecht, the Netherlands, were enrolled from October through May during 2005–2008. Patients were excluded if they had any of the following: asthma exacerbation, immunocompromised state, indication for antimicrobial drugs other than for LRTI, and repeated PICU admission for LRTI during the study period. Control group participants were children <18 years of age (median 2.2 years) who were admitted to the PICU from October 2005 through March 2006 for reasons other than LRTI.

Clinical data were obtained by using standardized forms to extract data from electronic charts. Underlying illnesses were defined as chronic pulmonary disease, congenital heart disease, immunodeficiency, malignancy, neurologic disease, or gastrointestinal disease (*8*). To assess the severity of illness, we used the lowest ratio during the first 24 hours of the partial pressure of oxygen in arterial blood (PaO_2)_ to the inspired oxygen fraction (FiO_2_). These ratios were acquired from the Pediatric Intensive Care Evaluation database, which contains validated clinical data for all Dutch PICU admissions.

Nasopharyngeal aspirates were collected from all patients in the LRTI group as part of the investigation of their illnesses. In the control group, nasopharyngeal aspirates were taken from intubated patients, and throat swabs were taken from extubated children as part of routine surveillance to identify transmission of respiratory syncytial virus (RSV). Because RSV surveillance was conducted as part of normal patient care, patient consent/ethical approval was not needed, according to the Medical Ethical Research Council of our institution.

Specimens from patients in the LRTI group were initially examined for RSV, influenza viruses, parainfluenza viruses, adenoviruses, rhinoviruses, coronaviruses, human metapneumovirus, and *Mycoplasma pneumoniae* by using real-time PCR as previously described (*1*,*9*,*10*). Specimens from patients in the control group were initially examined for RSV. All samples were retrospectively tested for HBoV, KIPyV, and WUPyV also by using real-time PCR as previously described (*11*,*12*). After nucleic acid extraction using the MagNA Pure LC 1.0 nucleic acid isolation system (Roche Diagnostics, Rotkreuz, Switzerland), amplification was carried out in a 25-μL reaction mixture on a 7500 Fast Real-Time PCR System (Applied Biosystems, Foster City, CA, USA). Positive controls for the KIPyV and WUPyV PCR were provided by S. Bialasiewicz and T.P. Sloots, University of Queensland, Queensland, Australia, and the positive control for HBoV was provided by T. Allander, Karolinska Institute, Stockholm, Sweden. Internal control viruses were used to monitor efficient extraction and amplification. Real-time PCR results were expressed in cycle threshold (Ct) values. Ct values are inversely correlated with viral load; i.e., low Ct values indicate high viral loads.

Of 90 LRTI patients enrolled, 78 (86.7%) had sufficient material stored for HBoV, KIPyV, and WUPyV testing. Eighty-eight control patients were enrolled, of which 83 (94.3%) had sufficient material stored to be included. Table 1 provides patients’ demographic and clinical characteristics. The main clinical conditions of control patients were cardiac disease requiring surgery (33.7%), trauma (8.4%), sepsis (8.4%), and upper respiratory tract infection (8.4%). A total of 57 (68.7%) nasopharyngeal aspirates and 26 (31.3%) throat swabs from the 83 patients who had sufficient samples were tested.

**Table 1:** Demographic and clinical characteristics for PICU patients with LTRIs and for controls, the Netherlands, 2005–2008*

Characteristics of patients	LRTI group, n = 78	Control group, n = 83
Sex, no. (%)		
M	43 (55.1)	44 (53.0)
F	35 (44.9)	39 (47.0)
Nasopharyngeal aspirates, no. (%)	78 (100.0)	57 (68.7)
Mechanical ventilation, no. (%)	76 (97.4)	79 (95.2)
Age, mo, median (IQR)	1.5 (4.2)	26.1 (134)
PaO_2_/FiO_2_, mm Hg, median (IQR)	130 (74.3)	250 (206)
Time on ventilator, d, median (IQR)	9 (5)	4 (9)
Time in hospital, d, median (IQR)	10 (6)	6 (11)

*PICU, Pediatric Intensive Care Unit, Wilhelmina Children’s Hospital, Utrecht, the Netherlands; LRTI, lower respiratory tract infection; IQR, interquartile range; PaO_2_/FiO_2_, ratio of the partial pressure of oxygen in arterial blood to the inspired oxygen fraction.

In LRTI patients, HBoV was found in 4 (5.1%) and WUPyV in 2 (2.6%) of the 78 patients. No samples tested positive for KIPyV. Table 2 shows Ct values and clinical characteristics for LRTI patients whose samples were positive as well as for controls whose samples were positive for these viruses. Other respiratory viruses were found in 70 (89.7%) of the 78 children. RSV was found in 52 (66.7%), influenza viruses in 3 (3.8%), parainfluenza viruses in 2 (2.6%), adenoviruses in 4 (5.1%), rhinoviruses in 20 (25.6%), coronaviruses in 6 (7.7%), human metapneumovirus in 5 (6.4%), and *Mycoplasma pneumoniae* in 1 (1.3%) of the patients. Multiple respiratory viruses were found in 3 of the 4 LRTI patients with HBoV infection and in both patients with WuPyV infection (Table 2). One patient had a single infection with HBoV (i.e., no other virus was detected). This patient was born at 31 weeks of gestational age and had a history of a grade IV idiopathic respiratory distress syndrome. She was admitted to the PICU at 19 months of age with a severe LRTI. Bacterial throat and blood cultures remained negative.

**Table 2:** Clinical characteristics of PICU patients with LRTIs and of PICU controls whose samples tested positive for HBoV, KIPyV, or WUPyV infections, the Netherlands, 2005–2008*

Patient no.	Diagnosis	Virus (Ct value)	Sample type	Sex	Age, mo	Immunocompromised	Other underlying disease	Length of stay, d	Other viruses
LRTI group									
1	LRTI	HBoV (39.4)	NPA	F	13	No	Pulmonary dysplasia (home mechanical ventilation)	10	Adeno, hMPV
2	LRTI	HBoV (15.0)	NPA	F	9	No	IRDS, PVL	10	Adeno
3	LRTI	HBoV (16.6)	NPA	M	13	No	Recurrent wheezing	5	RSV
4	LRTI	HBoV (15.0)	NPA	F	19	No	IRDS	7	None
5	LRTI	WUPyV (19.6)	NPA	M	1	No	None	11	RSV
6	LRTI	WUPyV (34.1)	NPA	F	41	No	Mitochondrial encephalopathy	18	Influenza
Control group									
7	Sepsis	HBoV (36.5)	NPA	F	27	No	None	7	NA
8	Infectious meningitis	HBoV (34.7)	NPA	F	100	No	22Q11 deletion (cardiac and neurologic disease)	3	NA
9	URTI	HBoV (37.9)	NPA	M	41	No	Spinal muscular atrophy	74	NA
10	Observation after brain biopsy	HBoV/ KIPyV (32/39)	NPA	F	127	Yes	AML, BMT, Brochiolitis obliterans	46	NA
11	VSD closure	WUPyV (33.8)	Throat swab	F	15	No	VSD	2	NA
12	Septic shock	WUPyV (34.4)	NPA	M	40	No	None	19	NA
13	Cardiac malformation	KIPyV (34.1)	NPA	F	4	No	Cardiac malformation	14	NA
14	Septic shock	KIPyV (23.1)	NPA	M	152	Yes	AML, aplasia	11	NA
15	ALTE	KIPyV (26.7)	NPA	M	2	No	None	8	NA

*PICU, Pediatric Intensive Care Unit, Wilhelmina Children’s Hospital, University Medical Center Utrecht, the Netherlands; LRTI, lower respiratory tract infection; HBoV, human bocavirus; KIPyV, KI polyomavirus; WUPyV, WU polyomavirus; Ct, cycle threshold; NPA, nasopharyngeal aspirate; Adeno, adenovirus; hMPV, human metapneumovirus; RSV, respiratory syncytial virus; IRDS, idiopathic respiratory distress syndrome; PVL, periventricular leukomalacia; NA, not assessed; URTI, upper respiratory tract infection; AML, acute myeloid leukemia; BMT, bone marrow transplant; VSD, ventricular septal defect; ALTE, apparent life-threatening event.

In the control group, HBoV was found in samples from 4 (4.8%) patients, KIPyV was found in 4 (4.8%), and WUPyV was present in 2 (2.4%) samples from the 83 patients whose samples could be tested. One patient was found to have a co-infection with HBoV and KIPyV.

Median Ct values for HBoV, KIPyV, and WUPyV combined were 18.1 (interquartile range [IQR] 20.4) for the LRTI group and 34.4 (IQR 5.1) for the control group (p = 0.09; Figure). The Ct values indicate that, on average, the viral load in the LRTI group might be higher than in the control group.

**Figure:**
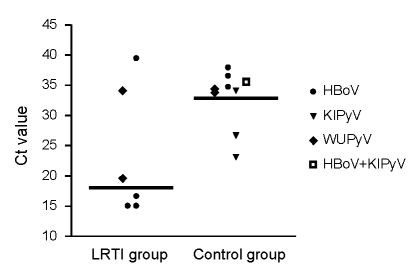
Cycle threshold (Ct) values of lower respiratory tract infection (LRTI) and control patients with human bocavirus (HBoV), KI polyomavirus (KIPyV), and WU polyomavirus (WUPyV) infections. LRTI patients are those admitted to the Pediatric Intensive Care Unit, Wilhelmina Children’s Hospital, University Medical Center Utrecht, the Netherlands; control patients are patients admitted to the Pediatric Intensive Care Unit with other diagnoses. Horizontal bars represent group medians (difference 16.3 Ct, p = 0.09).

## Conclusions

In the present study, the prevalences of HBoV, KIPyV, and WUPyV in PICU patients with LRTI (n = 78) or without LRTI (n = 83) were similar (5.1%, 0%, 2.6%; and 4.8%, 4.8%, 2.4%, respectively). Most HBoV- and KIPyW-positive LRTI patients were co-infected with other viruses. One LRTI patient with a HBoV single infection was identified. In this patient, HBoV was present in a high quantity.

Two limitations of our study deserve further discussion. First, LRTI patients were younger than controls (LRTI, 100% <5 years; controls, 40% >5 years). However, the positivity rates for HBoV, KIPyV, and WUPyV in control children <5 years were similar to rates of control children >5 years of age (6/49 vs. 3/34, respectively). Hence, the influence of this limitation is likely minor. Studies have also shown that the highest incidence of KIPyV/WUPyV infection occurs in children ≈1 year of age, slightly older than the children in our LRTI group (13–15). The young age of the LRTI group may have resulted in a lower than expected positivity rate for this group. Second, all LRTI patients had nasopharyngeal aspirates taken; however, 68.7% of controls had provided nasopharyngeal aspirates. HBoV and KIPyV/WUPyV infections were more common in controls who had nasopharyngeal aspirate samples taken than in those who had throat swab samples taken (8/57 vs. 1/26). This difference in positivity for nasopharyngeal aspirates strengthens our conclusion that these viruses are not found more frequently in PICU children with LRTI.

Sampling errors make precise quantification of viral loads difficult. Nevertheless, in the LRTI group, low Ct values, which indicate high viral loads, were found in nasopharyngeal samples taken from 3 patients infected with HBoV (Ct ≈ 15) and from 1 patient infected with WUPyV (Ct = 19). Ct values found in nasopharyngeal samples from patients in the control group were much higher, 32–39. A possible explanation for this difference is that high viral loads in the young LRTI population represent symptomatic primary infection, whereas the low viral load in the older controls might represent asymptomatic long-term shedding. Further studies are needed to show the clinical implications of infections with these viruses.

Prevalences of HBoV, KIPyV, and WUPyV infections in children in the PICU is low (≈<5% for LRTI patients and controls), and these agents are unlikely to be a major cause of LRTI at the PICU. However, HBoV might be pathogenic in some PICU patients because 1 person with a HBoV single infection in a high quantity was identified. Further studies using quantitative viral detection are needed to investigate the probability that HBoV, KIPyV, and WUPyV represent etiologic agents of LRTI.
